# Irisin and Bone: From Preclinical Studies to the Evaluation of Its Circulating Levels in Different Populations of Human Subjects

**DOI:** 10.3390/cells8050451

**Published:** 2019-05-14

**Authors:** Graziana Colaianni, Lorenzo Sanesi, Giuseppina Storlino, Giacomina Brunetti, Silvia Colucci, Maria Grano

**Affiliations:** 1 Department of Emergency and Organ Transplantation, University of Bari, 70124 Bari, Italy; graziana.colaianni@libero.it (G.C.); lorenzo.sanesi89@gmail.com (L.S.); g.storlino@gmail.com (G.S.); 2 Department of Basic Medical Sciences, Neuroscience and Sense Organs, Section of Human Anatomy and Histology, University of Bari, 70124 Bari, Italy; giacomina.brunetti@uniba.it (G.B.); silviaconcetta.colucci@uniba.it (S.C.)

**Keywords:** irisin, osteoporosis, sarcopenia

## Abstract

Almost four years after the discovery of the anabolic action of irisin on bone in mice, ample clinical evidence is emerging in support of its additional physiological relevance in human bone. Irisin inversely correlates with sclerostin levels in adults with prediabetes and with vertebral fragility fractures in post-menopausal women. Furthermore, in athletes we observed a positive correlation between irisin and bone mineral density at different anatomical sites. Our group also described a positive association between serum irisin and bone status in healthy children and multivariate regression analysis showed that irisin is a stronger determinant of bone mineral status than bone alkaline phosphatase. In children with type 1 diabetes mellitus, serum irisin concentrations are positively associated with bone quality and with glycemic control following continuous subcutaneous insulin infusion. Additionally, our in vitro studies suggest the existence of a negative interplay between PTH and irisin biology and these results were also supported by the observation that post-menopausal women with primary hyperparathyroidism have lower levels of irisin compared to matched controls. In this review, we will focus on recent findings about circulating level of irisin in different populations of human subjects and its correlation with their bone status.

## 1. Introduction

### 1.1. The Myokine Irisin

Skeletal muscle activation is a key aspect that improves quality of life in individuals who engage in physical activity because of stimulation of energy metabolism and synchronous reinforcement of bone and muscle mass. Large prospective studies have shown that running and walking activities reduce mortality by 20–40% [1,2] and slow down the progression of aging and related conditions including osteoporosis, diabetes and obesity [3].

Lately, a growing body of evidence suggests that muscle talks to bone not just through mechanical signals, but also via a finely tuned network of molecules termed myokines [4]. They are produced and released by myocytes in response to skeletal muscle contraction [5] and exert their systemic effects at picomolar concentrations [6]. Widely known representatives of the myokine family are myostatin, interleukin 15 (IL-15) and irisin.

The myokine irisin is cleaved from its precursor fibronectin type III domain-containing protein 5 (FNDC5) and secreted into the bloodstream. Irisin has been shown to stimulate white adipose tissue (WAT) to adopt a brown adipose tissue-like phenotype through increasing cellular mitochondrial density and expression of uncoupling protein-1. This trans-differentiation process triggers thermogenesis, increasing energy expenditure and improving glucose homeostasis [7]. However, own in-vivo studies suggest that the primary target organ of irisin is the skeleton rather than WAT. Thus, a weekly irisin dose 35 times lower than that required for the browning of WAT increases cortical bone mass and bone mechanical properties in mice [8].

### 1.2. Irisin in Mice

The skeletal effects of intermittently administered irisin have been studied in healthy mice and osteoporotic murine models. Treatment of healthy mice with recombinant irisin improves bone mass and its geometric and biomechanical properties compared to mice treated with vehicle [8]. Furthermore, administration of irisin partially prevents the development of disuse-induced osteoporosis and muscular atrophy in “hind-limb suspended” mice, a murine model which mimics adverse effects on musculoskeletal system caused by prolonged bed rest, physical immobility and microgravity exposure in humans [9,10]. Cortical and trabecular BMD, bone volume fraction (BV/TV) loss and Fractal Dimension are preserved in irisin treated mice subjected to musculoskeletal unloading [11]. As a potential mechanism through which irisin mediates its effects on bone, molecular studies showed reduction of sclerostin and increase of osteoprotegerin in unloaded mice treated with irisin that reach level of normally ambulating control mice, whereas the unloading increased and decreased their expressions, respectively [11]. The protective skeletal effects of irisin are accompanied by a preservation of muscle mass, fiber size and the expression of myosin Type II (MyHC II). Also, the expression of nuclear respiratory factor 1 (NRF1) and mitochondrial transcription factor A (TFAM) at control levels suggests that irisin treatment can prevent mitochondrial dysfunction during musculoskeletal unloading [11].

### 1.3. Irisin in Humans

In humans, irisin correlates inversely with serum sclerostin levels [12] and in post-menopausal women it is negatively associated with vertebral fragility fractures [13,14]. Furthermore, in athletes a positive association of irisin with BMD and bone strength has been found [15]. In soccer players we could demonstrate a positive correlation between irisin and BMD at different anatomical sites [16]. Our group has also described a positive association between serum irisin and bone status in healthy children [17]. Multivariate regression analysis showed that irisin is a stronger determinant of bone mineral status than bone alkaline phosphatase [17]. In children with type 1 diabetes mellitus, serum irisin concentrations are positively associated with a better glycemic control and bone quality [18]. Moreover, our in vitro studies suggest the existence of a negative interplay between PTH and irisin biology and these results were also supported by lower levels of irisin observed in post-menopausal women with primary hyperparathyroidism compared to matched controls [19].

In this review, we will focus on these recent findings about circulating levels of irisin in different populations of human subjects and its correlation with their bone status.

## 2. Irisin and Sport Activity

Although it has been clearly established that irisin serum concentration increases in response to exercise, the influence of exercise intensity (high-intensity or continuous-moderate) on irisin secretion remained uncertain. Some data suggest a greater increase following acute exercise compared with aerobic exercise [20], with a higher magnitude following high-intensity exercise [21,22]. Additionally, other results showed that, although FNDC5 expression was increased in skeletal muscle after a 12-week training of combined aerobic and acute exercises, there was a paradoxical decrease in circulating irisin, which spiked temporally up only after acute exercise [23].

Aiming to understand a possible influence of rising levels of irisin on increased bone mass in athletes, we investigated the correlation between its serum levels and total and sub-regional BMD in Caucasian football players of the Italian championship. Our results showed a positive correlation between irisin and total body BMD. Furthermore, linear association was also detected at specific bone-sites such as the right arm, lumbar vertebrae and head [16]. We did not expect a positive association with BMD in the upper limbs, as these bone segments receive a low impact by mechanical load. However, this finding suggests that the effects of circulating irisin on bone mass are systemic, rather than specific to bone-sites where load applies.

Beside the type of sport activity, also a day-night fluctuation may affect irisin synthesis in humans. Anastasilakis et al. performed an observational, cross-sectional study on 122 healthy, young individuals divided in subgroups subjected to day-night rhythm, standardized meal ingestion and 30-min aerobic exercise. Authors reported a day-night rhythm of irisin secretion, with its lowest levels early in the morning and its peak levels around 9:00 pm. Moreover, although authors showed that irisin was not affected by previous physical activity habits, they found higher circulating levels after short-term aerobic exercise [24].

## 3. Irisin in Children

Early childhood and adolescence are periods of rapid bone growth and the achievement of peak bone mass is a crucial determinant of lifelong skeletal health [25,26,27]. Experimental evidence supports the idea that bone mass in childhood is a predictor of pediatric fractures and that the amount of bone mass achieved by skeletal maturity is one of the most important contributors to peak bone mass, which in turn is the major determinant of osteoporotic fractures in aging [28]. Few studies investigated the relationship between irisin and bone status during childhood. Soininen et al. showed that circulating irisin was one of the determinants of bone mineral density in 6-8 years old children [29]. In a population of healthy children aged 7–13 years, we investigated possible correlations of irisin with bone metabolic markers and bone mineral status. Our findings demonstrated that irisin levels were positively correlated with bone quality evaluated by quantitative ultrasonography (QUS) after adjustment for age [17]. Moreover, multiple linear regression analysis showed that irisin was the strongest positive determinant of the bone transmission time (BTT) Z-score, which reflects the bone characteristics without soft tissue interference [30,31,32,33,34].

Interestingly, we found that irisin was positively correlated with circulating osteocalcin, the osteoblast-derived protein, which has been recently defined the bone-hormone that enhances energy metabolism, skeletal muscle function and brain development [35]. We also observed a negative association between irisin and DKK1, one of the bone anabolic WNT pathway inhibitors [36], thus further supporting the positive link of irisin with bone status in these children. Overall these studies suggest that encouraging children to exercise might be one of the best non-pharmacologic strategies to preserve their bone health, and this could also apply to pediatric pathologic conditions. For instance, type 1 diabetes mellitus (T1DM) patients have decreased BMD and a fracture risk six times higher than general population [37]. Bone loss can begin in childhood at the onset of diabetes, resulting in lower peak of bone mass and high risk for osteoporotic fractures in adult age and in elderly individuals [38]. We investigated irisin correlation with bone status in 96 children diagnosed with childhood type 1 diabetes mellitus (T1DM), further divided with 56 children receiving multiple insulin daily injections (MDI) and 40 children on continuous subcutaneous insulin infusion (CSII). In these patients, irisin negatively correlated with glycated hemoglobin (HbA1c%) and years of diabetes, and positively with BTT-Z-score and osteocalcin. Notably, we detected higher levels of irisin in CSII than MDI patients, indicating a strong positive association between irisin and the better glycemic control given by the pump device which closely mimics physiological pancreatic functions and improves the bone health status of these patients [18].

## 4. Irisin in Metabolic Syndrome

Metabolic syndrome (MetS) is a clinical condition in which a set of risk factors, including obesity, hypertension, dyslipidemia, hyperglycemia and insulin resistance, predispose to cardiovascular diseases, diabetes and osteoporosis [39,40]. Several studies have shown that MetS can be reversed, particularly by implementing lifestyle changes including recommendation to increase physical activity and adherence to a healthy diet for losing weight [41,42]. Aiming to understand the effects of a controlled diet restriction on circulating irisin levels, we analyzed 163 subjects with MetS that were randomized to one of three diets: Mediterranean diet (MD), Low Glycemic Index diet (LGID) and Low Glycemic Index Mediterranean diet (LGIMD). LGIMD is high in monounsaturated fats and low in carbohydrate. LGID and LGIMD have high fiber content. Starch intake is low in LGIMD and high in MD [43]. At baseline, we observed lower levels of circulating irisin in patients with Mets respect to healthy controls. During the dietary regime period, irisin serum levels tended to increase in all diet groups but the difference became significant only in LGID diet after six months. Overall, a greater adherence to the diet corresponded to higher levels of irisin, indicating that the effect of different types of diet is not only dependent on the composition of foods, but also on the adherence over time [43]. However, since physical activity is the key determinant of irisin levels, there was a limitation in our study which did not include any controls for exercise level in patients undergoing a dietary regime. This is certainly a key point that deserves attention in future studies in order to assess the relationship between irisin and the benefits of diet associated with physical activity in patients with MetS.

Our study was the first 6-month randomized trial monitoring irisin serum levels and the adherence to the diet throughout the trial. However, two previous studies on irisin and MetS, which were based on shorter dietary interventions, showed opposite results. A randomized, controlled trial, which compared the effects of two different 2-month-long hypocaloric dietary interventions, the control and RESMENA diets [44], reported that irisin levels were decreased similarly in both dietary groups during the follow up period. In another study, a group of 94 obese patients was enrolled in a weight loss program based on an 8-week hypocaloric diet with a weight maintenance follow-up. After 8 weeks of dietary treatment, irisin levels decreased in parallel with body weight reduction but returned to the baseline levels after 24 weeks in those patients regaining the lost weight [45].

## 5. Irisin in Primary Hyperparathyroidism

Patients affected by primary hyperparathyroidism (PHPT), an endocrine disorder characterized by excessive release of parathyroid hormone (PTH), experience reduction of BMD, particularly at the cortical site of the distal radius [46]. Biomolecular studies revealed that this chronic elevation of PTH increases receptor activator of nuclear factor-κB ligand (RANKL) and decreases osteoprotegerin (OPG) levels, leading to excessive activation of osteoclastogenesis [46]. Preclinical and clinical evidence has recently suggested a possible interaction between PHT and irisin, since both affect bone, muscle and adipose tissues, although in opposite ways. However, there were no studies until recently which investigated the possible interplay between these two hormones at the cellular level. Seeking to understand if irisin and PTH reciprocally influence their biological action, we analyzed FNDC5 expression in skeletal muscle cells treated with 1-34 PTH (Teriparatide). Our in vitro results showed that both short (3 hours) and long term (6 days) treatment with PTH negatively regulated FNDC5 mRNA and protein expression in myotubes by acting through the PTH receptor, which in turn activates Erk1/2 phosphorylation, most likely by increasing intracellular cAMP [19]. We also found that irisin serum levels were lower in post-menopausal women with PHPT compared to control subjects [19]. This finding supported results from other previous clinical investigations showing that irisin was inversely correlated with PTH in postmenopausal women with low bone mass [14] and in hemodialysis patients [47]. Moreover, our study also showed that irisin treatment decreases the expression of PTH receptor in osteoblasts, suggesting that this myokine may exert its anabolic effect on bone not only by stimulating osteoblast formation and function, but also by reducing the action of PTH on these cells [19].

## 6. The Putative Receptor for Irisin on Bone Cells

**Figure 1:**
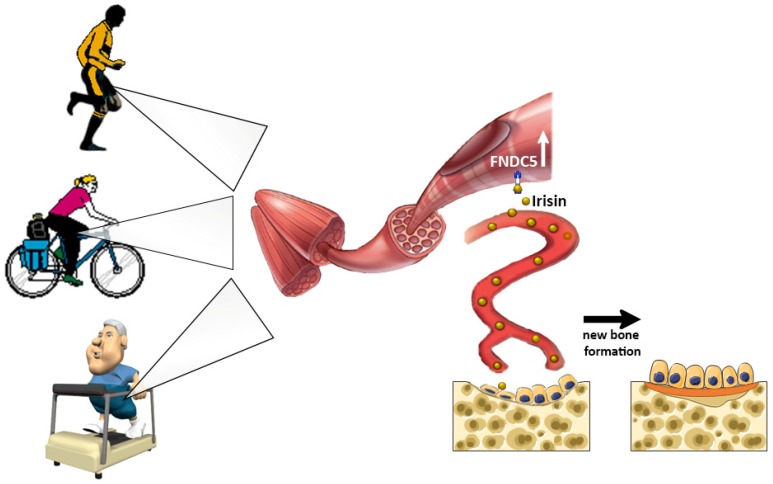
The myokine Irisin, produced by skeletal muscle and released into the circulation during physical activity, stimulates new bone formation.

Very recently Kim et al. identified integrin αV/β5 as the receptor for irisin on osteocytes [48]. The identification of irisin receptor might certainly improve the research on its downstream signaling cascades. However, authors showed that, although its highest affinity is for integrin αV/β5, irisin also binds to other integrin complexes. On the other hands, integrin αV/β5 has specific affinity for other ligands, such as osteopontin, bone sialoproteins and vitronectin [49], raising concerns about the unique intracellular signal pathway of irisin *in vivo*. Authors also provided evidence that irisin treatment upregulates the expression of sclerostin in osteocyte-like cells (MLO-Y4) and increases serum sclerostin levels in a dose dependent manner when injected in continuous manner once a day for 6 days in mice [48]. These results are apparently in contrast with our previous findings in unloading mice treated with rec-irisin [11] and with human studies reporting an inverse correlation between circulating irisin and sclerostin levels [12]. Furthermore, Kim and colleagues showed that FNDC5 global deletion in mice leads to reduced circulating levels of RANKL and increased femoral trabecular bone volume fraction. These mice were resistant to ovariectomy-induced bone loss through inhibition of osteoclastic bone resorption and osteolytic osteocytes [48]. These unfavorable effects of irisin on bone would be in contrast with the beneficial role on the skeleton exerted by muscle contractions (i.e., physical exercise) and hence by irisin secretion. However, authors explained possible differences with previous studies by using the paradigm of the PTH, which exerts both anabolic and catabolic effects on the skeleton depending on the intermittent or continuous administration, respectively [46]. Therefore, chronic high irisin levels could promote bone catabolism (i.e., increase sclerostin levels), while lower and discontinuous or null irisin levels, as in FNDC5 knockout mice, might be anabolic for bone. Therefore, an intermittent irisin pulse, as occurs during exercise, might transiently induce bone remodeling, leading to an overall favorable skeletal outcome [8,11] (Figure 1).

## 7. Conclusions

Almost four years after the discovery of the anabolic action of irisin on bone in mice [8], several clinical findings are also shedding light on its physiological significance in humans. The recent identification of its receptor will certainly facilitate new investigations to address whether the main functions of irisin identified in mice and in vitro assays, recapitulate the myokine’s physiology in humans. However, the questions of whether irisin acts on osteoblasts or osteoclasts through αV/β5 integrin and whether this binding results in similar or different intracellular responses still remain unanswered. Overall, since its discovery and for the relevance of numerous findings related to its role in bone metabolism, irisin has been often in the spotlight and raised debates and controversies. However, scientific consensus has unanimously established that irisin is a new key player in bone metabolism and its role is emerging as a possible therapeutic option to treat bone diseases.
